# Fat mass and obesity‐associated protein downregulation enhances N6‐methyladenosine methylation and drives ovarian cancer progression

**DOI:** 10.1002/ccs3.70049

**Published:** 2025-10-24

**Authors:** Xiaoling Wang, Dandan Wu, Chunxiao Li, Xiaomin Du

**Affiliations:** ^1^ Department of Obstetrics and Gynecology The First Hospital of Quanzhou Affiliated of Fujian Medical University Quanzhou China

**Keywords:** FTO, Ki67, m6A methylation, ovarian cancer, proliferation, xenograft

## Abstract

Ovarian cancer remains a major threat to women's health due to difficulties in early detection and limited treatment options. In this study, we investigate the role of FTO (fat mass and obesity‐associated protein), a key demethylase involved in N6‐methyladenosine (m6A) RNA modification, in the progression of ovarian cancer. Bioinformatics analysis of public datasets, along with validation in clinical samples, revealed that FTO expression was significantly lower in ovarian cancer tissues compared to normal controls. Functional assays demonstrated that FTO downregulation was associated with enhanced proliferation, migration, and invasion of ovarian cancer cells, which coincide with elevated global m6A methylation levels. Conversely, overexpression of FTO in vitro and in vivo significantly inhibited these tumorigenic phenotypes and suppressed tumor growth in a mouse xenograft model. Mechanistic studies demonstrated that FTO is localized in both the nucleus and cytoplasm and that its tumor‐suppressive effects are mediated, at least in part, through modulation of Ki67 expression. Together, these findings highlight FTO as a critical negative regulator of ovarian cancer progression and underscore the potential of targeting m6A methylation pathways as a therapeutic target. This research provides novel insights into the epitranscriptomic regulation of ovarian cancer and lays the groundwork for FTO‐based therapeutic development.

## Graphical abstract

FTO downregulation elevates global m6A methylation, enhancing ovarian cancer malignancy. Bioinformatics and experimental analyses revealed that the fat mass and obesity‐associated protein (FTO) was significantly downregulated in ovarian cancer tissues and cell lines. Loss of FTO increased global m6A methylation, promoting tumor cell proliferation, migration, and invasion, whereas FTO overexpression reversed these effects both in vitro and in vivo. Mechanistically, FTO modulated m6A methylation–dependent regulation of proliferation‐associated proteins such as Ki67, thereby suppressing tumor growth in xenograft models. This study identifies FTO as a key negative regulator of ovarian cancer progression and highlights the therapeutic potential of targeting the m6A methylation pathway.

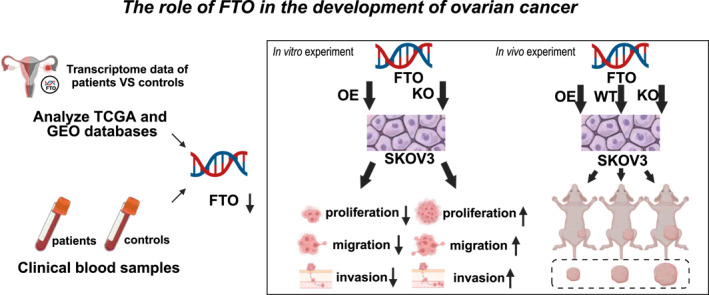

## 1 INTRODUCTION

Ovarian cancer is a highly malignant gynecologic tumor that poses a significant threat to women's health due to its high incidence and poor prognosis, making it a serious global public health concern.^1^, ^2^, ^3^ Although substantial advances have been made in the diagnosis and treatment of ovarian cancer over the past few decades, early detection remains difficult.^4^, ^5^, ^6^ Therefore, there is an urgent need to identify novel therapeutic targets and gain a deeper understanding of the molecular mechanisms underlying ovarian cancer pathogenesis.

N6‐methyladenosine (m6A) methylation, the most prevalent internal modification in eukaryotic mRNA, has recently gained attention as a key epigenetic regulator in cancer biology.^7^, ^8^, ^9^ This dynamic and reversible modification influences numerous cellular processes, including transcription regulation, RNA stability, splicing, and translational efficiency.^10^ Emerging evidence suggests that aberrant m6A methylation contributes to tumor development and progression by modulating cancer cell proliferation, invasion, and metastasis.^11^, ^12^, ^13^, ^14^ Given its central role in cancer biology, elucidating the function of m6A methylation in ovarian cancer may offer valuable insights into disease mechanisms and provide a foundation for the development of m6A‐targeted therapeutic strategies.

Fat mass and obesity‐associated protein (FTO), a key m6A RNA demethylase, plays a pivotal role in regulating m6A methylation dynamics.^15^, ^16^, ^17^ However, the expression patterns and functional roles of FTO in ovarian cancer remain poorly understood.^18^, ^19^ To address this gap, we first performed bioinformatics analyses using transcriptomic datasets from ovarian cancer patients and normal controls obtained from public databases, including The Cancer Genome Atlas (TCGA) and the Gene Expression Omnibus (GEO), with a focus on the differential expression of FTO.^20^, ^21^, ^22^ To validate these findings, we collected peripheral blood samples from clinical ovarian cancer patients and healthy controls. Global m6A RNA methylation levels were quantified using the EpiQuik m6A RNA Methylation Quantification Kit. In parallel, FTO expression was examined via reverse transcription quantitative PCR (RT‐qPCR) and western blot analysis.

Overall, this study aimed to elucidate the role and underlying mechanisms of FTO in ovarian cancer development. Our results demonstrated that FTO expression was significantly downregulated in ovarian cancer samples compared to normal controls and was strongly associated with increased cellular proliferation, migration, and invasion. In vivo experiments further confirmed that FTO overexpression markedly suppressed tumor initiation and growth in mouse models. These findings identify FTO as a critical regulatory factor in ovarian cancer pathogenesis and highlight its potential as a therapeutic target. By uncovering the tumor‐suppressive function of FTO, our study provides a scientific foundation for the development of FTO‐based diagnostic and therapeutic strategies, which may ultimately enhance early detection and treatment outcomes in ovarian cancer.

## 2 MATERIALS AND METHODS

### 2.1 Animal ethics statement

All animal experiments in this study were conducted in strict accordance with internationally recognized guidelines for the ethical use of animals in research. The experimental protocols were reviewed and approved by the Institutional Animal Care and Use Committee of our institution. Efforts were made to minimize animal suffering by ensuring appropriate housing, environmental enrichment, and humane care. All procedures were performed under proper anesthesia and analgesia to ensure animal welfare throughout the study.

### 2.2 Clinical research ethics statement

The collection and use of clinical specimens were conducted in compliance with institutional and national ethical regulations. Ethical approval was obtained from the relevant institutional review board. All participants were fully informed about the study's objectives, procedures, potential risks, and benefits. Informed consent was obtained from each participant prior to enrollment. The confidentiality and security of all patient data were strictly maintained, and clinical data handling adhered to applicable data protection regulations.

### 2.3 Bioinformatics analysis

#### 2.3.1 Database query and data retrieval

Gene expression datasets relevant to ovarian cancer were retrieved from the GEO database (https://www.ncbi.nlm.nih.gov/geo/) using the keyword “ovarian cancer.” Three datasets were selected for analysis: GSE18520 (GPL570 platform), which includes 10 normal ovarian tissue samples and 53 ovarian cancer tissue samples; GSE26712 (GPL96 platform), containing 10 normal and 185 cancer tissue samples; and GSE190688 (GPL11154 platform), comprising 5 normal and 6 ovarian cancer tissue samples.

#### 2.3.2 Data processing and analysis

All datasets were normalized and processed using the R programming language and the “limma” package. Differential gene expression analysis was performed using thresholds of |logFC| > 0.5 and adjusted *p*‐value < 0.05. Genes associated with ovarian cancer were identified using the GeneCards database (https://www.genecards.org/). A Venn diagram was generated using the “VennDiagram” R package to illustrate overlapping differentially expressed genes (DEGs) across the datasets. These shared genes were subsequently subjected to enrichment analysis. To investigate potential protein interactions and identify m6A‐related regulatory genes, we utilized the STRING database (https://cn.string‐db.org/) for functional protein–protein interaction (PPI) network analysis.

### 2.4 GO and KEGG enrichment analysis

To functionally annotate and identify pathways associated with DEGs, we performed Gene Ontology (GO) and Kyoto Encyclopedia of Genes and Genomes (KEGG) enrichment analyses using the DAVID Bioinformatics Resources (version 6.8, https://david.ncifcrf.gov/). Upregulated and downregulated genes were analyzed separately. The gene input format was standardized Gene Symbol, and the background was set to the complete Homo sapiens genome.

GO analysis encompassed three domains: biological process, molecular function, and cellular component. KEGG analysis was conducted to identify potential signaling pathways. A *p*‐value < 0.05 was used as the threshold for statistical significance.

After enrichment analysis, result files were downloaded and imported into the R environment for visualization. Bar plots and bubble plots were generated using the R packages clusterProfiler (version 4.0.5, https://bioconductor.org/packages/release/bioc/html/clusterProfiler.html) and ggplot2 (version 3.3.3, https://ggplot2.tidyverse.org/), respectively, to illustrate the degree of enrichment and statistical significance of each functional term or pathway. Multiple hypothesis testing was corrected using the Benjamini–Hochberg method.

### 2.5 Detection of m6A levels in ovarian cancer

A total of 20 ovarian cancer patients (tumor Stages I–III) who had undergone chemotherapy were enrolled in the case group. Surgical ovarian tissue samples were collected, embedded in paraffin, and stored at −80°C for subsequent analysis. Total RNA was extracted using TRIzol reagent from patient‐derived ovarian cancer tissues, the normal human ovarian epithelial cell line IOSE80, and the ovarian cancer cell line SKOV3 for the comparative analysis of m6A methylation levels. Clinical information for all patients and healthy controls is summarized in Table S1. Quantification of m6A RNA methylation was performed using the EpiQuik^TM^ m6A RNA Methylation Quantification Kit (Cat. No. P‐9005‐48, EpiGentek). The assay involved loading negative control RNA, positive control RNA, and 200 ng of sample RNA into designated wells, followed by incubation with capture and detection antibodies. After a series of incubation and washing steps, the absorbance was measured at 450 nm using a microplate reader. The relative m6A level was calculated using the following formula: m6A% = [(ODs − ODnc)/*S*]/[(ODpc − ODnc)/*P*] × 100%; where *S* = 200 ng and *P* = 1 ng.^23^

### 2.6 In vitro cell culture

#### 2.6.1 Cell lines and culture conditions

Three cell lines were used in this study: normal human ovarian epithelial cells (IOSE80, iCell‐h112), ovarian cancer cells (SKOV3, iCell‐h195), and HEK293T cells (iCell‐h237) as a transfection tool line. All cell lines were obtained from iCell Bioscience Inc. Cells were cultured in RPMI 1640 medium (GIBCO) supplemented with 10% fetal bovine serum (FBS, Cat. No. 12484028, GIBCO), and maintained in a humidified incubator at 37°C with 5% CO_2_. Once cells reached 80%–90% confluency, they were passaged using 0.25% trypsin‐EDTA solution. Cells were subcultured for up to 10 passages. For cryopreservation, cells at optimal confluency were resuspended in freezing medium containing 10% DMSO and stored in liquid nitrogen.^23^

#### 2.6.2 Morphological observation

Cell morphology and growth status were routinely monitored using an inverted phase‐contrast microscope (Olympus Corporation). Observations focused on cell shape, density, and confluency to ensure healthy proliferation and to confirm cells remained within the recommended passage range.

### 2.7 FTO knockout and overexpression

#### 2.7.1 Vector construction

FTO gene knockout was achieved using the CRISPR/Cas9 system with the vector pSpCas9 (BB)‐2A‐Puro (PX459)‐sgRNA‐FTO.^24^ The specific single guide RNA (sgRNA‐FTO) sequence used for targeting FTO is listed in Table S2. For gene overexpression, a lentiviral vector FTO‐pCDH‐CMV‐MCS‐EF1‐CopGFP was designed and synthesized by OBiO Technology.^25^

#### 2.7.2 Cell transfection and infection

SKOV3 ovarian cancer cells were seeded into 6‐well plates and transfected when they reached 70%–90% confluency. Transfection was performed using Lipofectamine 3000 reagent (Invitrogen, Cat. No. L3000150) according to the manufacturer's protocol. For knockout experiments, 2500 ng of either the FTO‐targeting plasmid (FTO‐KO group) or a nontargeting control plasmid (NC‐KO group) was transfected into each well. The DNA was mixed with 5 μL of P3000 reagent and 5 μL of Lipofectamine 3000, incubated at room temperature for 10–15 min, and then added to the cells under serum‐free conditions to enhance transfection efficiency. The plates were incubated at 37°C in a humidified incubator with 5% CO_2_. For overexpression experiments, 293T packaging cells were used for lentiviral production. Cells were seeded at 5 × 10^5^ cells/well in 6‐well plates and transfected with the FTO‐pCDH‐CMV‐MCS‐EF1‐CopGFP construct (FTO‐OE group) or the corresponding control plasmid (NC‐OE group), along with packaging plasmids pSPAX2 and pMD2.G, using Lipofectamine 3000 as described above. Viral supernatants were collected after 48 h and used to infect SKOV3 cells at a multiplicity of infection (MOI) of 20. SKOV3 cells were plated in 6‐well plates and infected when they reached 70%–90% confluency. Cells were then maintained at 37°C, 5% CO_2_ for viral transduction.^26^

#### 2.7.3 Selection of stable cell lines

Forty‐eight hours post‐transfection, drug selection was initiated using puromycin (Sigma, Cat. No. 540222) at a final concentration of 2 μg/mL in RPMI 1640 medium. The puromycin concentration was gradually increased in a stepwise manner (2/4/6/8/10 μg/mL) over 2 weeks to establish stable gene‐edited cell lines. Knockout and overexpression efficiencies were confirmed by real‐time quantitative PCR (RT‐qPCR) and western blot analyses.

### 2.8 Cell functional experiments

#### 2.8.1 Cell proliferation assay

Cell proliferation was assessed using the Cell Counting Kit‐8 (CCK‐8; Dojindo Molecular Technologies, Cat. No. CK04). Cells were seeded in 96‐well plates at a density of 1 × 10^3^ cells/well in 100 μL of RPMI 1640 medium containing 10% FBS. After 1–5 days of incubation, cell adhesion was confirmed under a microscope. Once cells were fully attached (defined as 0 h), 10 μL of CCK‐8 reagent was added to each well at 0, 24, 48, and 72 h. Plates were incubated for an additional 2 h at 37°C, and absorbance was measured at 450 nm using a microplate reader (BioTek). Growth curves were subsequently plotted.^27^

#### 2.8.2 Colony formation assay

Cells in the logarithmic growth phase were digested with trypsin, resuspended into single cells, and seeded into 6‐well plates at 1000 cells/well. After culturing for 14 days under standard conditions, visible colonies were fixed with 4% paraformaldehyde for 15 min and stained with 0.1% crystal violet for another 15 min. Colonies with a diameter >200 μm were counted manually.^28^

#### 2.8.3 Transwell migration and invasion assays

Transwell chambers (Millipore, Cat. No. CLS3398) were used to assess cell migration and invasion in 24‐well plates. For invasion assays, the lower surface of the membrane was precoated with Matrigel. A total of 3 × 10^4^ cells were resuspended in serum‐free DMEM with 1% FBS and seeded into the upper chambers. For migration assays, the Matrigel coating was omitted. The lower chambers were filled with medium containing 20% FBS as a chemoattractant. After 24 h of incubation, nonmigrated cells were removed, and cells on the lower membrane surface were fixed and stained with crystal violet. Migrated/invaded cells were counted under a microscope.^27^

### 2.9 RT‐qPCR

Total RNA was extracted from cultured cells using TRIzol reagent (Invitrogen) according to the manufacturer's instructions. cDNA was synthesized from 1 μg of total RNA using a reverse transcription kit (Part No. 4366597, Invitrogen). Quantitative real‐time PCR was performed using the SYBR GreenER^TM^ qPCR SuperMix Universal Kit (Part No. 11762500, Invitrogen). Relative gene expression was calculated using the 2^−ΔΔCt^ method, with GAPDH as the internal reference gene. Each experiment was performed in triplicate, and results were averaged from at least three independent biological replicates.^25^ Primer sequences are listed in Table S2.

### 2.10 Western blot

Total cellular proteins were extracted using M‐PER® Mammalian Protein Extraction Reagent (Thermo, Cat. No. 78505). Equal amounts of protein (30 μg) were separated via SDS‐PAGE on 10% or 6% polyacrylamide gels and transferred onto 0.2 μm polyvinylidene difluoride (PVDF) membranes (Millipore, Cat. No. ISEQ00010). Membranes were blocked with QuickBlock^TM^ blocking buffer (Beyotime Biotechnology, Cat. No. P0220) and incubated overnight at 4°C with rabbit anti‐FTO (Abcam, Cat. No. ab126605) and rabbit anti‐β‐actin (Abcam, Cat. No. ab8227) antibodies, both diluted 1:1000. After three washes with Tris‐buffered saline containing 0.1% Tween 20 (TBST, Cat. No. ST671), membranes were incubated with HRP‐conjugated secondary antibody (1:5000, Abcam, Cat. No. C31460100) at room temperature for 1 h. Following additional TBST washes, bands were visualized using enhanced chemiluminescence reagents and band intensities were quantified using ImageJ software.^25^

### 2.11 Cell apoptosis detection using flow cytometry

SKOV3 cells were harvested and washed twice with ice‐cold PBS. Apoptosis was assessed using the Annexin V‐FITC/PI Apoptosis Detection Kit following the manufacturer's instructions. Briefly, cells were incubated with 5 μL of Annexin V‐FITC and 5 μL of PI at room temperature for 15 min in the dark. Apoptotic cells were then analyzed using a BD FACSymphony A3 flow cytometer (BD Biosciences).^29^

### 2.12 Establishment and intervention of a subcutaneous tumor model in nude mice

#### 2.12.1 Animal acquisition and grouping

A total of 40 female BALB/c nude mice (6 weeks old; Cat. No. F100104) were purchased from BIOMICE. Mice were housed under specific pathogen‐free conditions and allowed free access to food and water. After 1 week of acclimatization, the mice were randomly assigned into four groups (*n* = 10 per group): FTO knockout control group (NC‐KO), FTO overexpression control group (NC‐OE), FTO knockout group (FTO‐KO), and FTO overexpression group (FTO‐OE).

#### 2.12.2 Establishment of tumor model

SKOV3 cells with stable FTO knockout or FTO overexpression were used for tumor implantation. A total of 5 × 10^6^ cells in 100 μL of sterile PBS were injected subcutaneously into the right flank of each mouse. Control groups were injected with an equal number of vector‐only control cells. All procedures were performed under sterile conditions to minimize infection and ensure reliable tumor establishment.

#### 2.12.3 Tumor growth monitoring

Tumor progression was monitored weekly. Tumor size was measured using digital calipers, and the tumor volume was calculated using the formula: *V* = 1/2 × L × W^2^. In addition, in vivo tumor development was monitored using a small animal imaging system (IVIS Spectrum, PerkinElmer) to visualize tumor burden and assess progression dynamically.

#### 2.12.4 Sample collection and analysis

Two weeks after tumor implantation, mice were euthanized in accordance with institutional ethical guidelines. Tumors and relevant organs were harvested for downstream analysis. Histopathological examination and immunohistochemistry (IHC) staining were performed to evaluate tumor cell proliferation, invasiveness, and senescence‐related markers.^30^

### 2.13 IHC staining

#### 2.13.1 Tissue processing

Excised tumor tissues were fixed, embedded in paraffin, and sectioned. The sections were then deparaffinized, rehydrated through a graded ethanol series, and treated with methanol containing 3% hydrogen peroxide for 20 min to quench endogenous peroxidase activity.

#### 2.13.2 Antigen retrieval and blocking

Antigen retrieval was performed using a water bath and an antigen retrieval buffer. To prevent nonspecific binding, tissue sections were blocked with normal goat serum (Product No. 0060‐01, Shanghai Haoran Biotechnology Co. Ltd.).

#### 2.13.3 Staining procedure

Primary antibodies, including rabbit anti‐FTO (1:1000, Abcam, Product No. ab126605) and rabbit anti‐Ki67 (Abcam, Product No. ab15580), were applied to the sections and incubated overnight at 4°C. The following day, after rinsing with PBS, sections were incubated with a horseradish peroxidase (HRP)‐conjugated secondary antibody for 20 min at 37°C. Color development was performed using a DAB chromogenic substrate kit, followed by counterstaining with hematoxylin, dehydration through a graded ethanol series, clearing with xylene, and mounting with neutral resin.

#### 2.13.4 Microscopic observation and quantification

Stained sections were observed and imaged using a light microscope (e.g., Olympus). Five random high‐power fields were selected per section, and 100 cells per field were manually counted. Quantitative analysis was performed using image analysis software such as ImageJ.^31^

### 2.14 Statistical analysis

All data processing and statistical analyses were conducted using R (version 4.2.1) and the RStudio integrated development environment (version 4.2.1). Data preprocessing was performed using Perl (version 5.30.0), and network visualization was conducted using Cytoscape software (version 3.7.2). Quantitative results were expressed as mean ± standard deviation (SD). Group comparisons were conducted using independent samples *t*‐tests (for two groups) or one‐way ANOVA (for comparisons among three or more groups). Two‐way ANOVA was applied for time‐series analyses. Where applicable, Bonferroni correction was used for post hoc multiple comparisons. A *p*‐value < 0.05 was considered statistically significant.

## 3 RESULTS

### 3.1 Gene expression analysis reveals DEGs in ovarian cancer

**FIGURE 1:**
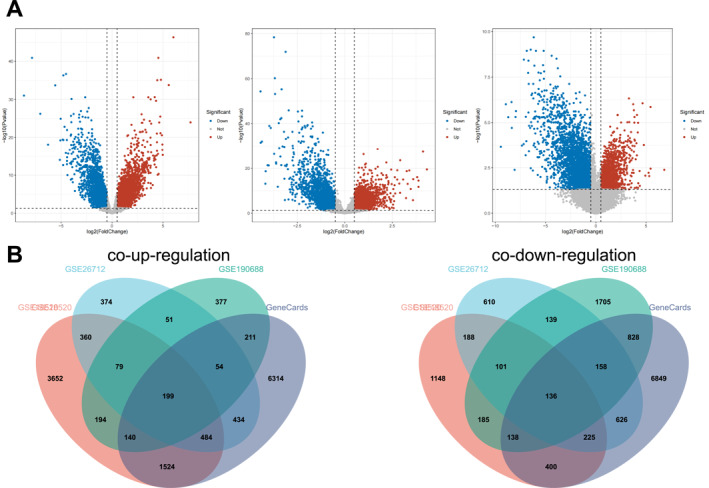
Volcano plots of differentially expressed genes and Venn diagram of gene intersection. (A) Volcano plots of differentially expressed genes in GSE18520, GSE26712, and GSE18520; red dots represent significantly upregulated genes, whereas blue dots indicate significantly downregulated genes (threshold: |logFC| > 0.5, adjusted *p* < 0.05). (B) Venn diagram showing the overlap of DEGs from the three datasets and ovarian cancer‐related genes retrieved from the GeneCards database (relevance score > 1). The intersecting gene sets form the basis for subsequent enrichment and interaction analyses. DEGs, differentially expressed genes.

We performed transcriptomic analysis using three independent datasets related to ovarian cancer to identify DEGs. In the GSE18520 dataset, 6633 genes were significantly upregulated and 2521 genes were downregulated in ovarian cancer tissues compared to normal controls. Similarly, analysis of the GSE26712 dataset revealed 2035 upregulated and 2183 downregulated genes, whereas the GSE190688 dataset showed 1305 upregulated and 3390 downregulated genes in ovarian cancer tissues (Figure 1A). To further refine the list of relevant DEGs, we performed an integrative analysis by intersecting the commonly upregulated and downregulated genes across the three datasets with genes associated with ovarian cancer from the GeneCards database (Relevance Score > 1). The overlapping genes are illustrated in the Venn diagram in Figure 1B.^32^ This approach yielded 199 consistently upregulated genes (Table S3) and 136 consistently downregulated genes (Table S4), which were considered as significantly dysregulated in ovarian cancer.

### 3.2 Enrichment of DEGs in biological pathways related to ovarian cancer

**FIGURE 2:**
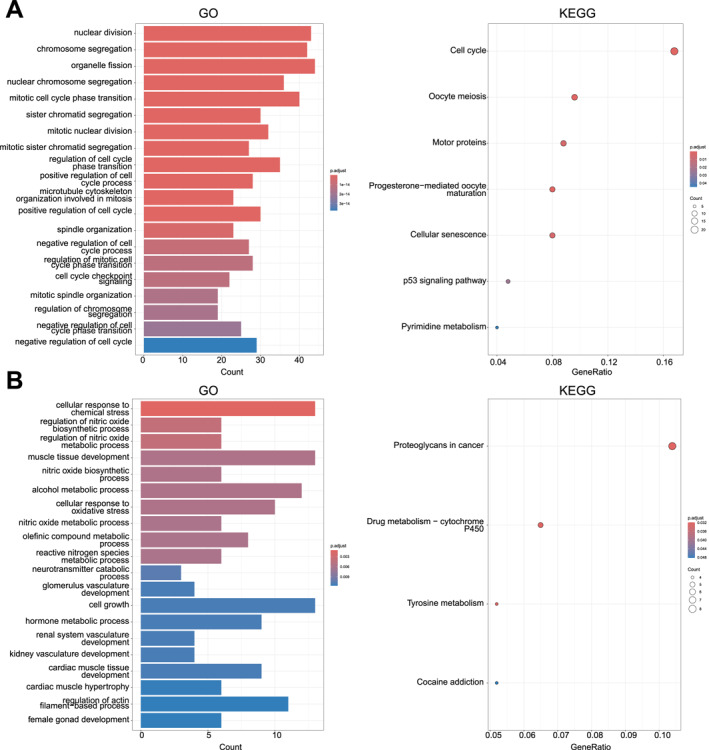
Enrichment analysis of DEGs. (A) GO and KEGG enrichment analysis results of upregulated genes in ovarian cancer; (B) GO and KEGG enrichment analysis results of downregulated genes in ovarian cancer. DEGs, differentially expressed genes; GO, Gene Ontology; KEGG, Kyoto Encyclopedia of Genes and Genomes.

To explore the biological significance of the DEGs identified in ovarian cancer, we conducted GO and KEGG enrichment analyses for both upregulated and downregulated gene sets (Figure 2). GO enrichment analysis of the upregulated genes revealed significant enrichment in biological processes related to nuclear division and chromosome segregation (Table S5). KEGG enrichment analysis further indicated that these genes were predominantly enriched in pathways regulating the cell cycle (Figure 2A and Table S6). In contrast, downregulated genes were primarily enriched in GO terms associated with the cellular response to chemical stress and nitric oxide biosynthesis and metabolism (Table S7). KEGG pathway analysis revealed a significant enrichment of these genes in pathways related to aberrant protein glycosylation (Figure 2B and Table S8). These findings collectively highlight the functional relevance of the dysregulated genes and their potential contribution to the pathophysiology of ovarian cancer.

### 3.3 PPI network construction and identification of core genes

**FIGURE 3:**
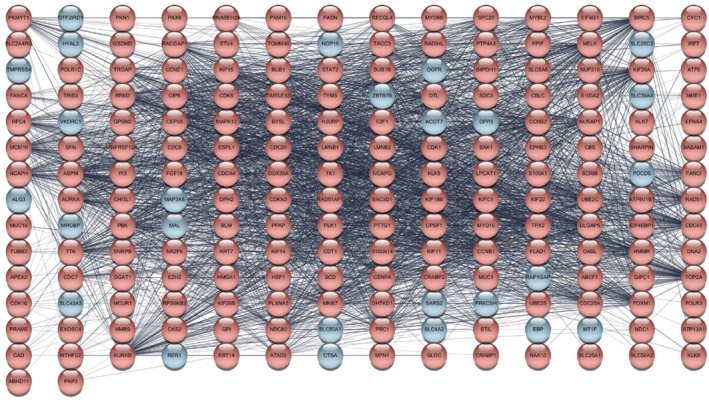
PPI network of upregulated genes in ovarian cancer. The PPI network was constructed based on upregulated genes in ovarian cancer. Each node represents a protein; node color indicates connectivity, with blue nodes representing a degree value of 0. PPI, protein‐protein interaction.

**FIGURE 4:**
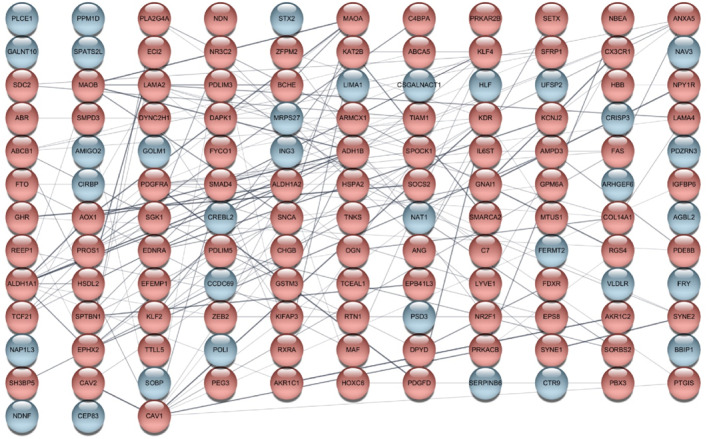
PPI network of downregulated genes in ovarian cancer. The PPI network was constructed based on downregulated genes in ovarian cancer. Each node represents a protein; blue nodes indicate a degree value of 0. PPI, protein–protein interaction.

To identify key regulatory genes in ovarian cancer, we constructed PPI networks separately for upregulated and downregulated DEGs using interaction degree values to evaluate connectivity. Nodes (genes) with a degree value of zero were excluded to eliminate isolated proteins (Figures 3 and 4). After filtering, a total of 172 upregulated genes and 101 downregulated genes were retained in the respective PPI networks.

### 3.4 Potential role of downregulated FTO in m6A methylation regulation during ovarian cancer progression

**FIGURE 5:**
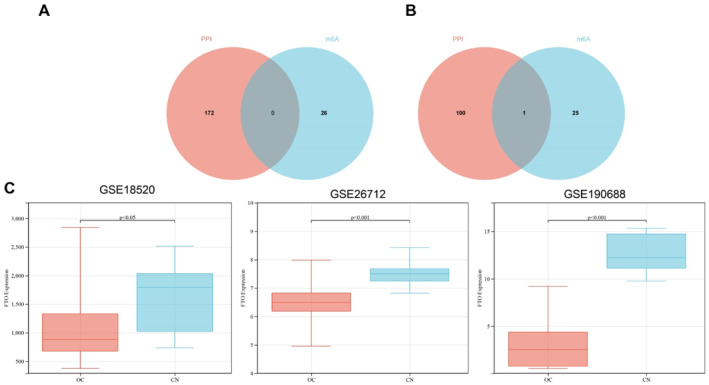
Venn diagram of intersection between PPI analysis results and m6A‐related genes and expression differences of FTO in ovarian cancer. (A) Venn diagram of intersection between upregulated genes and m6A‐related genes after PPI filtering; (B) Venn diagram of intersection between downregulated genes and m6A‐related genes after PPI filtering; (C) differential expression of FTO between normal and ovarian cancer samples across multiple datasets (GSE18520: ovarian cancer *n* = 53, NC *n* = 10; GSE26712: ovarian cancer *n* = 185, NC *n* = 10; GSE190688: ovarian cancer *n* = 6, NC *n* = 5). Statistical significance was determined using an independent samples *t*‐test. FTO, fat mass and obesity‐associated protein; m6A, methyladenosine; NC, negative control; PPI, protein‐protein interaction.

m6A is a dynamic and reversible RNA modification that plays a critical role in regulating RNA metabolism and has recently gained considerable attention in cancer research, including ovarian cancer.^33^ To explore the potential involvement of m6A regulators in ovarian cancer, we performed an intersection analysis between the genes identified through PPI network analysis and 26 well‐characterized m6A‐related genes. This analysis revealed FTO as a key candidate (Figure 5), suggesting that its downregulation may contribute to ovarian cancer progression through dysregulation of m6A methylation.

### 3.5 FTO regulates m6A methylation and promotes proliferation, invasion, and migration of ovarian cancer cells

**FIGURE 6:**
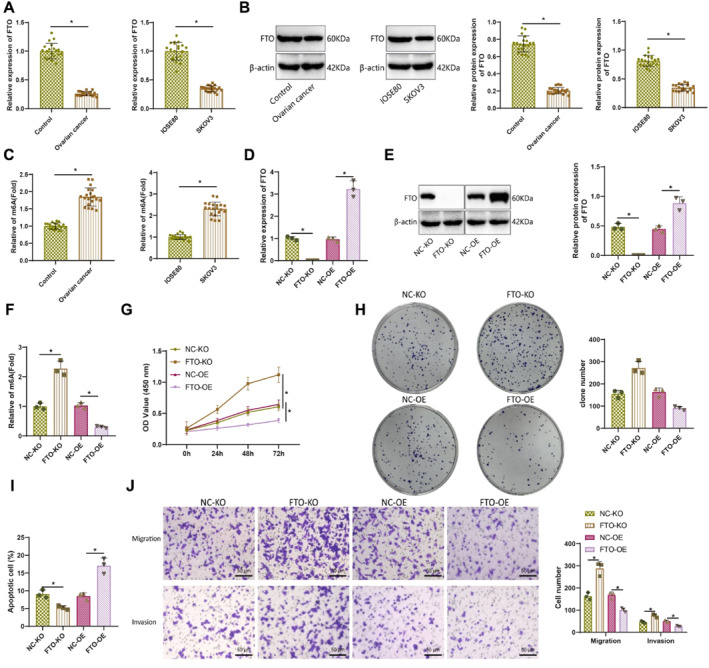
Downregulation of FTO in ovarian cancer cells promotes cell proliferation, migration, and invasion by upregulating m6A levels. (A) Changes in FTO mRNA expression in 20 cases of ovarian cancer tissues and SKOV3 ovarian cancer cell line were detected using RT‐qPCR; (B) changes in FTO protein expression in 20 cases of ovarian cancer tissues and SKOV3 ovarian cancer cell line were detected using WB; (C) the levels of m6A methylation in 20 cases of ovarian cancer tissues and SKOV3 ovarian cancer cell line were measured using the EpiQuik m6A RNA methylation quantification assay kit; (D) changes in FTO mRNA expression in SKOV3 ovarian cancer cell line after FTO knockout or overexpression were detected using RT‐qPCR; (E) changes in FTO protein expression in SKOV3 ovarian cancer cell line after FTO knockout or overexpression were detected using WB; (F) the levels of m6A methylation in SKOV3 ovarian cancer cell line after FTO knockout or overexpression were measured using the EpiQuik m6A RNA Methylation Quantification Assay Kit; (G) the proliferation ability of SKOV3 ovarian cancer cell line after FTO knockout or overexpression was measured using CCK8; (H) the colony formation ability of SKOV3 ovarian cancer cell line after FTO knockout or overexpression was detected using the colony formation assay; (I) the apoptosis of SKOV3 ovarian cancer cell line after FTO knockout or overexpression was detected using flow cytometry; (J) the migration and invasion abilities of SKOV3 ovarian cancer cell line after FTO knockout or overexpression were measured using the transwell assay. All cell experiments were performed with three biological replicates, * represents *p* < 0.05. FTO, fat mass and obesity‐associated protein; m6A, methyladenosine.

To evaluate FTO expression in ovarian cancer, we measured mRNA and protein levels in patient‐derived ovarian cancer tissues and matched control samples, as well as in the normal ovarian epithelial cell line IOSE80 and the ovarian cancer cell line SKOV3, using RT‐PCR and western blot. Both mRNA and protein levels of FTO were significantly reduced in ovarian cancer tissues and SKOV3 cells compared to controls (Figure 6A,B).

Next, we assessed global m6A methylation levels in total RNA isolated from the same tissues and cell lines. A significant increase in m6A methylation was observed in ovarian cancer tissues compared to normal controls, and similarly, m6A levels were significantly higher in SKOV3 cells than in IOSE80 cells (Figure 6C).

To further investigate the regulatory role of FTO in m6A methylation, we manipulated FTO expression in SKOV3 cells via overexpression (FTO‐OE) and CRISPR/Cas9‐mediated knockout (FTO‐KO). RT‐PCR and western blot analyses confirmed successful gene editing: FTO expression was significantly downregulated in the FTO‐KO group and upregulated in the FTO‐OE group relative to their respective controls (Figure 6D,E). Measurement of m6A methylation levels revealed that FTO knockout significantly increased, whereas FTO overexpression significantly decreased m6A levels in SKOV3 cells (Figure 6F).

The proliferative, apoptotic, invasive, and migratory capacities of SKOV3 cells were assessed under different treatment conditions. Results from the clonogenic assay, CCK‐8 proliferation assay, and apoptosis assay demonstrated that, compared to the NC‐KO group, the FTO‐KO group exhibited significantly enhanced proliferative and colony‐forming abilities, accompanied by a marked reduction in apoptotic activity. Conversely, the FTO‐OE group displayed significantly reduced proliferation and clonogenic potential compared to the NC‐OE group, along with a notable increase in apoptosis (Figure 6G–I). Moreover, transwell migration and invasion assays revealed that FTO‐KO cells had significantly increased migratory and invasive capabilities compared to the NC‐KO group. In contrast, FTO‐OE cells exhibited significantly reduced migration and invasion relative to the NC‐OE group (Figure 6J).

In summary, our findings indicate that FTO expression is significantly downregulated in ovarian cancer cells, contributing to elevated m6A methylation levels. This epigenetic alteration promotes enhanced proliferation, invasion, migration, and clonogenic potential while simultaneously suppressing apoptotic signaling.

### 3.6 The enhancing role of FTO in regulating ovarian cancer development: Modulating M6A methylation and proliferation proteins

**FIGURE 7:**
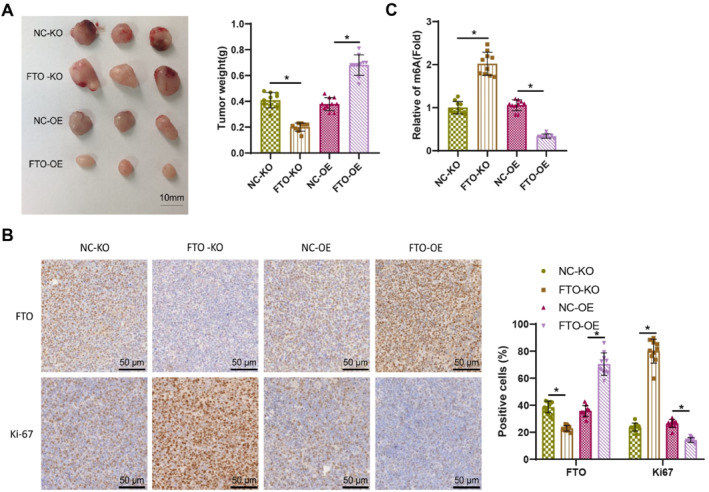
Downregulated expression of FTO promotes the growth of ovarian cancer tumors in mice through the upregulation of m6A. (A) The tumorigenic ability and tumor weight of the SKOV3 ovarian cancer cell line were evaluated in a subcutaneous tumor model in mice after FTO knockout or overexpression (*N* = 10); (B) the expression of FTO and Ki67 in different tumors of the subcutaneous tumor model in mice after FTO knockout or overexpression was determined using immunohistochemistry (*N* = 10); (C) the m6A methylation levels in different tumors of the subcutaneous tumor model in mice after FTO knockout or overexpression were measured using the EpiQuik m6A RNA Methylation Quantification Assay Kit (*N* = 10). * represents *p* < 0.05. FTO, fat mass and obesity‐associated protein; m6A, methyladenosine.

To further validate the regulatory role of FTO in ovarian cancer progression, we established a subcutaneous xenograft tumor model by injecting SKOV3 cells with either FTO knockout or FTO overexpression constructs into nude mice. Tumor growth analysis revealed a significant increase in tumor volume in the FTO‐KO group compared to the NC‐KO group. In contrast, the FTO‐OE group exhibited a marked reduction in tumor weight compared to the NC‐OE group (Figure 7A). IHC staining of tumor tissues further supported these findings. In the FTO‐KO group, FTO protein expression was significantly reduced, whereas the expression of Ki67, a well‐known marker of cell proliferation, was markedly increased compared to the NC‐KO group. Conversely, the FTO‐OE group demonstrated elevated FTO protein levels and significantly reduced Ki67 expression relative to the NC‐OE group (Figure 7B). Consistent with these observations, m6A methylation analysis revealed that FTO knockout led to a significant increase in m6A methylation levels in tumor tissues compared to the NC‐KO group. Conversely, FTO overexpression significantly reduced m6A methylation in tumor samples compared to the NC‐OE group (Figure 7C).

Collectively, these results demonstrate that FTO suppresses ovarian cancer progression in vivo by increasing m6A methylation and the expression of proliferation‐associated proteins, particularly Ki67.

## 4 DISCUSSION

As research on ovarian cancer—a highly malignant tumor that poses a significant threat to women's health—continues to advance, increasing attention has been directed toward the role of m6A RNA modification in tumorigenesis and cancer progression.^34^, ^35^ The primary aim of this study was to explore the diagnostic value of peripheral blood m6A methylation levels in ovarian cancer and to elucidate the molecular mechanisms involving the RNA demethylase FTO. Using bioinformatics analyses of transcriptomic data derived from ovarian cancer patients and normal controls in publicly available databases such as TCGA and GEO,^20^, ^21^, ^22^ we identified FTO as a significantly DEG in ovarian cancer. These findings provide potentially valuable insights for improving both the diagnosis and treatment of ovarian cancer.^36^, ^37^, ^38^

Although previous studies have suggested a role for FTO in various cancers, its specific function in ovarian cancer remains incompletely understood.^35^, ^39^ Our findings not only corroborate the dysregulation of FTO in ovarian cancer but also build upon existing knowledge by clarifying its association with m6A methylation levels and oncogenic behaviors such as cell proliferation, migration, and invasion.^18^, ^19^

Importantly, we observed a significant downregulation of FTO in ovarian cancer tissues and cell lines, which was closely correlated with increased m6A methylation and enhanced tumor cell aggressiveness. These results align with studies in other cancer types where FTO acts as a tumor suppressor by regulating m6A modification.^40^, ^41^, ^42^ In summary, our study supports the hypothesis that FTO plays a critical inhibitory role in the development and progression of ovarian cancer, primarily through its modulation of m6A methylation. These findings not only advance our understanding of the epitranscriptomic landscape in ovarian cancer but also highlight FTO as a promising biomarker and potential therapeutic target for future clinical applications.^43^, ^44^, ^45^

Through in vivo experiments using a murine xenograft model, we further validated the tumor‐suppressive role of FTO in ovarian cancer development.^35^, ^46^ Overexpression of FTO significantly inhibited tumor formation and reduced tumor growth, consistent with previous findings on the anticancer potential of FTO.^47^ These results support the notion that FTO may serve as a promising therapeutic target, offering a novel direction for ovarian cancer treatment.

We also examined the subcellular localization of FTO and its correlation with ovarian cancer cell proliferation. IHC analysis revealed that FTO is predominantly localized in both the nucleus and cytoplasm of tumor cells,^48^, ^49^, ^50^ supporting its multifaceted regulatory role. Although our findings are largely consistent with existing studies, minor variations in FTO localization suggest that further research is required to fully elucidate its intracellular distribution and context‐specific functions.

Based on our results, several conclusions can be drawn: FTO expression is significantly downregulated in ovarian cancer tissues compared to normal controls, and this reduction is closely associated with increased cell proliferation, migration, and invasion. Overexpression of FTO markedly inhibits tumorigenic behavior both in vitro and in vivo, whereas FTO knockout promotes ovarian cancer progression. FTO is primarily localized in the nucleus and cytoplasm, and co‐localization studies with the proliferation marker Ki67 suggest a potential regulatory interaction contributing to tumor cell proliferation. These findings provide novel insights into the molecular pathogenesis of ovarian cancer and establish a mechanistic link between m6A RNA methylation and tumor progression.

The scientific and clinical significance of this study lies in its identification of FTO as a key regulator of m6A methylation and tumor growth in ovarian cancer. By integrating transcriptome data with clinical tissue analysis and functional experiments, we demonstrated that low FTO expression correlated with poor tumor behavior and FTO restoration suppressed tumorigenesis in both cellular and animal models. In conclusion, our study highlights FTO as a critical epitranscriptomic regulator and a potential biomarker and therapeutic target in ovarian cancer. These findings open new avenues for the early diagnosis, prognostic assessment, and targeted therapy of ovarian cancer, addressing some of the most pressing challenges in current clinical management.

Despite the promising findings of this study, several limitations should be acknowledged. First, the relatively limited sample size may introduce bias and restrict the generalizability of the results. Second, although animal models provided important experimental evidence, the clinical relevance and translational validity of these findings require further verification in larger patient cohorts and real‐world clinical settings. Third, this study did not extensively investigate the underlying signaling pathways or downstream molecular mechanisms regulated by FTO. Previous studies have highlighted mechanistic roles of FTO in other cancers. For example, in cervical cancer, FTO regulates m6A methylation of ZEB1 and Myc, key oncogenes involved in cell proliferation and migration. Silencing FTO results in decreased expression of these targets, thereby suppressing tumor progression.^51^ In gastric cancer, FTO silencing reduces the expression of MOXD1, a gene associated with poor prognosis, and may influence cancer‐related pathways.^52^ These findings provide valuable directions for future mechanistic studies in ovarian cancer to determine whether similar regulatory axes are involved. In addition, the therapeutic potential of targeting FTO or m6A methylation was not addressed in this study. This remains an important area for further exploration, particularly in identifying drugs or genetic interventions that can modulate m6A‐related pathways for clinical benefits.

From a broader perspective, future research should aim to clarify the regulatory mechanisms of FTO and m6A methylation in ovarian cancer progression and explore their interactions with other signaling networks. Notably, FTO has been reported to play dual roles in cancer, acting as either an oncogene or tumor suppressor depending on the tumor type and cellular context. For instance, in colorectal cancer, overexpression of FTO and ALKBH5 suppresses proliferation both in vitro and in vivo, whereas their silencing enhances tumor growth.^53^ In contrast, in pancreatic neuroendocrine tumors, FTO promotes malignancy by enhancing APOE expression through FASN‐mediated lipid metabolism, suggesting an oncogenic role.^54^ Our current findings support a tumor‐suppressive function of FTO in ovarian cancer, consistent with the context‐dependent behavior reported in other malignancies.

**FIGURE 8:**
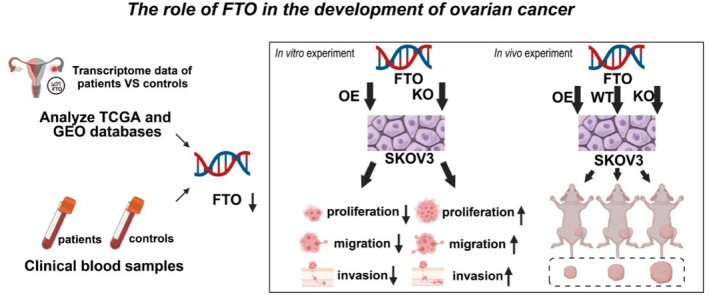
The mechanism of FTO in the development of ovarian cancer. FTO, fat mass and obesity‐associated protein.

Going forward, future studies should evaluate the therapeutic feasibility of targeting m6A pathways and FTO, including the identification of small molecules or biologics that can modulate FTO activity or expression. Moreover, clinical studies and trials are essential to assess the diagnostic and therapeutic value of FTO and m6A methylation in ovarian cancer, with the ultimate goal of developing more effective and personalized treatment strategies for patients (Figure 8).

## AUTHOR CONTRIBUTIONS

X. W. conceived and supervised the study. D. W. and C. L. performed the experiments and data analysis. X. D. contributed to clinical sample collection and bioinformatics analysis. X. W. and D. W. wrote the manuscript. All authors reviewed and approved the final version of the manuscript.

## CONFLICT OF INTEREST STATEMENT

The authors declare no conflicts of interest.

## ETHICS STATEMENT

All animal experiments were approved by the Animal Ethics Committee of The First Hospital of Quanzhou Affiliated of Fujian Medical University.

## Supporting information

Table S1

Table S2

Table S3

Table S4

Table S5

Table S6

Table S7

Table S8

## Data Availability

All data can be provided as needed.
