# Supplementary material for: Fat mass and obesity‐associated protein downregulation enhances N6‐methyladenosine methylation and drives ovarian cancer progression
Source: J Cell Commun Signal. 2025 Oct 24;19(4):e70049. doi: 10.1002/ccs3.70049 (PMC12552135; doi:10.1002/ccs3.70049)
Supplement: Supplementary file 1 — Table S1 [file CCS3-19-e70049-s005.docx]

**Table S1. Clinical information of ovarian cancer patients and corresponding volunteers**

| **Characteristic** | | **Value** | **Number of Samples** |
| --- | --- | --- | --- |
| Age (years) | Control | 62.3 ± 10.2 | 20 |
|  | Ovarian cancer | 61.3 ± 5.2 | 20 |
| BMI [kg/m2] | Control | 30.2 ± 5.7 | 20 |
|  | Ovarian cancer | 26.9 ± 5.7 | 20 |
| TNM stage | I | NA | 9 |
|  | II | NA | 6 |
|  | III | NA | 5 |
