# Supplementary material for: Fat mass and obesity‐associated protein downregulation enhances N6‐methyladenosine methylation and drives ovarian cancer progression
Source: J Cell Commun Signal. 2025 Oct 24;19(4):e70049. doi: 10.1002/ccs3.70049 (PMC12552135; doi:10.1002/ccs3.70049)
Supplement: Supplementary file 2 — Table S2 [file CCS3-19-e70049-s001.docx]

**Table S2. Gene editing primers and transfection sequences**

| **Target gene** | **Primer/transfection sequence type** | **Sequence** | **Explanation** |
| --- | --- | --- | --- |
| FTO | sgRNA sequence | 5'-CTACGAGCTGAAGCCGAGCG-3' | Specific editing of the FTO gene |
| NC | sgRNA sequence | 5'-AATTAGATGGTGATGTTAA-3' | Negative control for FTO gene-specific editing |
| FTO | RT-qPCR primer | F: 5'-AGGTGCCAGTCACGAATTG  -3' R: 5'-AGAGGCATCGAAGCATCATC  -3' | Validation of FTO knockout efficiency |
| GAPDH | RT-qPCR primer | F: 5'-ACAGTCAGCCGCATCTTCTT-3' R: 5'-GACAAGCTTCCCGTTCTCAG-3' | Reference gene for quantitative analysis |
